# Germ Diseases and Methods of Control

**Published:** 1920-07

**Authors:** C. E. A. Winslow


### GERM DISEASES AND METHODS OF CONTROL

BY C. E. A. WINSLOW, M.D.

The original tendency was to look outside for the causes of disease, to lay the blame on the swamps and the night air, and all sorts of external things. Gradually it was forced upon us that it was not in these outside things, but in the bodies of human beings themselves, that the danger lay. The danger lies in the transfer of bodily discharges from one human being to another, and the transfer must be a rather quick one if the germ is to be kept alive and virulent.

Then came the recognition of a new type of human being besides the sick person who might be dangerous—the carrier. In a great many of those diseases it was possible for the germ to live in the body of an individual without doing any harm, but capable after transfer to another person of producing active disease. Our object today, therefore, is to prevent the passage of discharges from one person to another, whether the first person be ill or well, because even if a person is well he may be a carrier of the germs of transmissible disease.

The vehicles by which disease germs pass from one person to another may be divided conveniently into three great classes—food, fingers and flies; food, including water and milk; flies, including all sorts of insects; and fingers, including direct contact.

Some diseases are easy to handle and others not. We know now, for example, that bubonic plague may be controlled. This disease broke out the last time in 1894, and spread to seaports all over the world, from Australia to New Orleans, to Scotland and San Francisco; yet in no place, except in India, did it gain a foothold, because we knew it spread from rats to men by the bite of a flea, and we were enabled to control it by a systematic campaign against the rat.

The particular pest of armies in every war all through history has been typhus fever. In the Japanese-Russian war there was a great outbreak. Typhus decimated Napoleon's army, and it was typhus that saved Constantinople from its enemies in the Balkan war. Now we know the disease is spread by the bite of the louse and that it is easily and effectively controlled.

You know the story of yellow fever; how in 1898 when the American army of occupation went into Cuba they found there had been 750 deaths a year from yellow fever in the city of Havana. They could not do anything to control this pestilence at first. You remember those four Americans, Reed, Carroll, Lazear and Agramonte, how they experimented upon themselves, and proved that yellow fever was caused by the bite of a particular mosquito. Carroll came down first, and then Lazear, who gave his life to the cause. One of the grandest things in the history of medicine was this calm facing of death from a terrible disease.

In March, 1901, Reed and his colleagues solved their problem, and the scourge of centuries was practically wiped out in a year. So, one by one the insect-borne diseases have been practically brought under control.

We do not have today any great epidemics of food-borne diseases since milk has been pasteurized and water purified. We can eliminate lice and mosquitoes, and we can purify water and pasteurize milk, but we can not pasteurize people, and they are the source of danger in contact-borne diseases. They remain the great causes of diphtheria and measles and pneumonia and influenza and diseases of that type. Material from one person's mouth gets into another person's mouth—that is the way these diseases spread. It is not pleasant, but it is so, and that is the fundamental thing you must remember in all these cases of colds and sore throat and pneumonia and kindred diseases.

In dealing with these contact-borne diseases in times past there was a quarantine at the state boundaries called "shotgun quarantine." The states adjoining would plant men with shotguns to prevent people from crossing the border. We saw something like that in the paralysis epidemic. This old method of quarantine was not very effective and it interfered with commerce and travel. Newer methods have now come into vogue—methods which we group under the heading of isolation. Quarantine is a rough method applied to groups of people. Isolation is applied to the particular individuals who are considered dangerous. A few years ago, for instance, a number of cases of cholera were brought into America when there was an epidemic in Italy, and the ships were actually detained only for two or three days while bacteriologists examined the discharges of each person to determine whether he was a carrier or not. Then they isolated the carriers and let the others go. Isolation is much less irksome and burdensome than quarantine, and it has been very successful in many cases. It must, however, include the carrier as well as the actual cases. Cerebro-spinal meningitis, for instance, is one of the serious diseases of military life, and when it breaks out all the men's throats are cultured, and those who are carriers of the dreaded germ of cerebro-spinal meningitis detected and isolated.

There are many diseases, however, in which we can not make isolation. Take measles, for instance. Measles is most contagious in the early stages when it can not be recognized as measles at all, and when it is considered as only cold in the head. Yet it is actually more dangerous at this stage than when the rash appears. It is difficult to deal with problems of that kind.

Again, take infantile paralysis. The trouble there is of a somewhat different nature. This disease is spread mainly by well carriers. In cerebro-spinal meningitis we can pick out the carriers, but in infant paralysis we can not pick them out, because the germ concerned can only be isolated by the use of monkeys, which can not be obtained in any number. This is a sufficiently professional gathering for me to confess openly that none of the things that any of the health departments did about infant paralysis did the least bit of good. They had to go through the motions and do the best they could; but it did not affect the prevalence of the disease in the least degree. As far as prevention goes, we can not do anything with infant paralysis, because it is spread by well carriers, and we can not detect those carriers.

With influenza the case is still more hopeless. We know almost nothing about this disease. We do not know how it is spread, or how to detect it in the dangerous stage. There is only one thing that helped—absolute quarantine. Institutions that were kept shut up tight were immune for a time, but the disease usually got in after awhile when the quarantine was relaxed. In one institution in Connecticut they escaped it entirely while the city all about was suffering, until early in January some children were sent down to the post-office, and then they caught it, and about 400 children in that institution came down with influenza.

There were marked differences in the number of deaths in different cities. Cities that were in generally good condition came off easily. Those that had all sorts of other diseases suffered severely. That was all one could say about it.

There are certain groups of diseases that have been completely conquered by sanitation; but about diseases like influenza and infant paralysis we have not enough information as yet. Then there are the diseases with which you deal, the sub-acute infections and focal infections. They are not directly traceable to infection from one person to another. They may be communicable, but slowly, as tuberculosis, not in an acute, definite form.

There is obviously little that the health authorities can do directly in dealing with the contact-borne diseases. The only way to handle them is by education, by changing the habits of the individual; personal education must take the place of police procedure. If we can make people understand that all these diseases come from getting into the mouth something filthy that has come from the mouth of some one else, we can do a great deal. We must acquire the aseptic sense, the instinct that keeps out of the mouth things that are not clean, in place of the normal instinct to put everything into the mouth, the instinct with which babies are born, or else are endowed soon after they are born.

The health officer, as a health officer, standing up in his might and issuing orders, serves a useful function; but he has done all he can. Now he must get down off his platform and talk to people individually and try to change their habits. He can not quarantine the early cases of disease, because he can not recognize them. It is only the individual father or mother or teacher or companion in the home or the factory who can recognize these acute communicable diseases in their very early stage. So in your field you must educate the people and train them to have their teeth cleaned and get the proper care they require. That is simply symptomatic of the greatly widening scope of education in the field of public health.

The health officer of today ought to be a man who thinks in statistical terms. He does not ask, "Is such and such a thing dangerous?" The private physician deals with one individual at a time, and he has to do everything he can for that individual. The health officer has a whole community to deal with and a limited amount of money; and he must say, "How many lives can I save for a dollar?" He sees that some of the things, like cleaning up the back yards and supervising the food stores, are not really going to save a great many lives. He sees that the control of communicable diseases, particularly tuberculosis and infant mortality and venereal diseases, are three of the great profitable lines of undertaking. Take tuberculosis, still accounting for one-quarter of all deaths in young adult life. We can deal with that partly by attending to the surroundings, by protecting men from bad environments, such as the dusty atmosphere of grinding shops, but we must work mainly by education in hygiene. The main thing is to change the people's daily habits of living.

Infant mortality is the greatest single line of activity we can pursue from the standpoint of results. The infant mortality rate is calculated as so many deaths under one year of age in proportion to one thousand births. In Russia before the war there were 250 deaths per thousand births. In the United States the figure is a little over 100 —I think 120. In New Zealand it is fifty. Only one baby in twenty in New Zealand dies before it reaches its first birthday, and that is the direct result of the work of infant welfare stations.

So the campaign against venereal diseases is partly a problem of providing clinics and treatment, but mainly an educational campaign.

Then take the great field of mental hygiene. We have heard a good deal about the war psychosis, commonly called shell shock, but it has nothing to do with shells. There are some people always well balanced to meet any situation. Others are excitable and flustered and worried, and things go hard with them; there is a different degree of balance and co-ordination. Whether a person can get along in a community or not is a measure of the balance of his mental equipment. In war a certain number who should have remained normal have been pushed over the line by overstrain; that is merely what war psychosis means. England and Canada did little about it. There are thousands and thousands of their men blind and disabled as a result. When America entered the war a little group of people in New York went right to Washington and persuaded the surgeon-general to organize a force to deal with the question. Of about ten thousand cases of war neurosis, how many do you think were found who can not be helped? Not more than one hundred. This wonderful result was achieved by mental first aid, by the efforts of a force of psychiatrists who treated these cases in the beginning, and prevented them from developing further.

There are cases of that kind in peace, just the same as in war. I heard this afternoon of such a case—the man happens to be a returning soldier, but his trouble only developed lately. There are people in every large city in

Connecticut who are acting a little bit queerly and getting out of touch; they say people do not like them, and everything is going wrong and they are beginning to be obsessed by one idea or another. That was just the position of those men who came out of the trenches. These people, if taken care of, will come out all right, but if they do not receive proper treatment at the beginning, the trouble will progress until some day the victim will kill somebody with an axe.

We turn almost wholly to women for the actual work of individual health education, I suppose because this kind of teaching requires patience and tact, and those are not particularly masculine qualities. We turn first of all to the nurses, and in many of these fields the public health nurse has become the central figure in the whole movement. We are trusting her to "carry the message to Garcia," to get the knowledge of the laws of health into the lives of the people who need it. The social worker, the psychiatric social worker—all kinds of helpers are being trained. We must give Dr. Fones the credit for this great movement here in Bridgeport, and for starting this new profession of dental hygiene which makes such a fine showing on its fifth anniversary. You are a particularly successful and valuable addition in this great force that we are sending out to cope with the problem of ignorance as a factor in disease.

Nothing we are doing is comparable to what we shall do. Think of the amount of money spent in training children's minds, and of the slight attention paid to the health of their bodies.

The physician has today no chance to treat most diseases in the early stages when they are most readily curable. He does not even know what their early stages are like. Some one said of a recent book on hardening of the arteries, that it read like the third volume of a three-volume novel.

We ought not to be building hospitals, and waiting for people to become ill and die, but we ought to work seriously on the prevention of these troubles. It is coming. I do not know whether it will come by health insurance, or by expansion of public clinics, or by teaching in schools, but it will come. Medical and dental service and nursing service—all these things must be socialized and used by the community in such an effective way that every baby and every child and every grown person shall have as a matter of course the hygienic instruction and the bodily care he needs. There are certain principles in hygiene we teach to everybody, things about food and air and exercise and rest that apply to everybody, but there are other things that apply to certain individuals. If a man has heart trouble, or a cancer, he needs special treatment based on physical examination. We should all be examined at regular intervals yearly, perhaps ultimately, and the definite specific advice needed should be given, and the treatment furnished, so the bodies of all the people may be kept in the right condition.

The work in Bridgeport is an inspiration to us all. All of us in public health work are grateful to Dr. Fones and to you, because you have shown the whole world what to do for the care of the teeth of school children. Do not forget, however, that your movement in oral hygiene is part of this general campaign for developing a system for the care of the bodies of all, so we shall reach a maximum of health, so we may all feel that "just to live, and move, and breathe, should be a delight." England has a national board of health and it will come here. There will be a wider campaign in public health than we have dreamed of in the past. With your influence there will come about ultimately the right scheme that will put the state care of health on a basis as wide and sound as that on which our system of state education rests today.—*Cosmos* for May, 1920.

